# A novel mutation of congenital nephrotic syndrome in a Slovenian child eventually receiving a renal transplant

**DOI:** 10.3325/cmj.2021.62.187

**Published:** 2021-04

**Authors:** Valentina Golob, Gregor Nosan, Sara Bertok, Maja Frelih, Emanuela Boštjančič, Rina Rus

**Affiliations:** 1 General Hospital Murska Sobota, Murska Sobota, Slovenia; 2 Department of Neonatology, Division of Pediatrics, University Medical Centre Ljubljana, Ljubljana, Slovenia; 3 Department of Endocrinology, Diabetes and Metabolic Diseases, Division of Pediatrics, University Medical Centre Ljubljana, Ljubljana, Slovenia; 4 Institute of Pathology, Faculty of Medicine, University of Ljubljana, Ljubljana, Slovenia; 5 Department of Nephrology, Division of Pediatrics, University Medical Centre Ljubljana, Ljubljana, Slovenia

## Abstract

Congenital nephrotic syndrome (CNS) is a rare disease defined as heavy proteinuria, hypoalbuminemia, hyperlipidemia, and edema presenting in the first three months of life. It is most commonly caused by mutations in the *NPHS1* gene associated with nephrotic syndrome type 1, also known as Finnish-type CNS, which is inherited in an autosomal recessive manner. Symptomatic treatment with intravenous albumins, vitamins, minerals, nutritional, and hormonal supplementation and treatment of complications are mandatory. Children refractory to the symptomatic treatment are recommended to undergo nephrectomy and renal replacement therapy, preferably renal transplantation. We report on a child with Finnish type CNS with a *NPHS1* mutation, which is the first case confirmed by genetic study in Slovenia. We showed for the first time that homozygous mutation c.2928-3del in the *NPHS1* gene caused exon 22 skipping, leading to a truncated protein and Fin-minor phenotype.

Congenital nephrotic syndrome (CNS) is a rare form of nephrotic syndrome, presenting in the first three months of life. In most cases, it is caused by monogenic mutations of structural proteins that form the glomerular filtration barrier in the kidneys, such as gene *NPHS1* (1).

Pathogenic variants in the *NPHS1* gene are associated with nephrotic syndrome type 1, also known as Finnish-type CNS, which is inherited in an autosomal recessive manner, with the mutations being homozygous or compound heterozygous. The syndrome is characterized by a severe steroid-resistant nephrotic syndrome apparent at birth, with rapid progression to end-stage renal failure (1,2).

We report on a child with Finnish-type CNS with a *NPHS1* mutation, which is the first case confirmed by a genetic study in Slovenia. The reported mutation was confirmed for the first time to be pathogenic.

## CASE REPORT

The patient was a girl born after 35 weeks gestation, with normal body measurements. On the first day of life, edema of the lower extremities was observed. Her mother has hereditary leyomiomatosis and renal cell cancer (HLRCC), with a mutation in gene *FH* (c.1189G>A;p.Gly397Arg), and essential thrombocythemia, with a mutation in gene *JAK2.* The mother's brother also had HLRCC and died from kidney carcinoma.

At the age of three days, blood tests revealed hypoalbuminemia, hypoproteinemia (30 g/L), and hyperlipidemia. Renal function was normal. Proteins in urine were 4+, with erythrocytes 3+. Perinatal infections were excluded. Abdominal ultrasound showed enlarged hyperechogenic kidneys.

Blood was taken for genetic analysis with next generation sequencing technologies, which revealed a previously unreported homozygous variant of uncertain significance – c.2928-3del, in the *NPHS1* gene. The diagnosis of the CNS of Finnish type was very likely. Both parents are heterozygous for this variant. The girl was also heterozygous for HLRCC, with a mutation in gene *FH*, previously found in the mother.

Because of massive loss of proteins (up to 11 656 g proteins per mol creatinine) and hypoalbuminemia (11-20 g/L), the patient received albumin parenterally once a day and 4 g/kg of proteins per day in her diet. She was administered indomethacin, later changed to ibuprofen due to vomiting, and captopril. Excessive proteinuria persisted despite treatment.

To maintain adequate diuresis, she received furosemide. Immunoglobulin supplements were given parenterally due to hypogammaglobulinemia.

**Figure 1:**
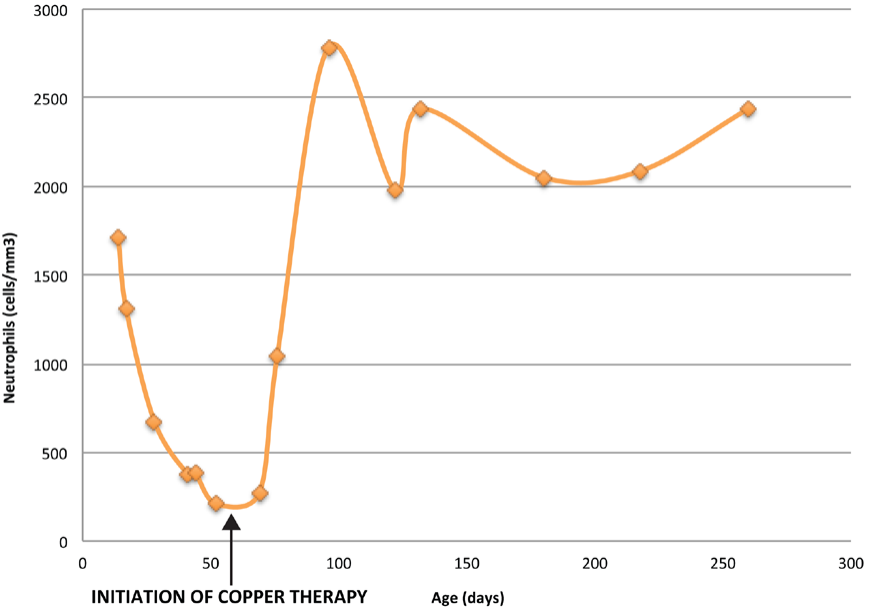
Improvement in neutrophil count after the initiation of copper therapy in a child with Finnish type congenital nephrotic syndrome with a *NPHS1* mutation

At the age of 1.5 months, anemia and severe neutropenia (378 cells/mm^3^) were observed. Low serum iron, transferrin, thyroid hormone, and erythropoietin levels were detected, so she received hormones, together with iron, vitamin, and mineral supplements. Additionally, the total copper serum level and ceruloplasmin were decreased, so oral supplementation with copper was started, up to 15 mg per day. During the copper therapy, the neutrophil count normalized (Figure 1).

A low antithrombin III level was observed. Because of the increased risk of thrombosis, she received acetylsalicylic acid and had no thromboembolic events.

She had no appetite and received a hypercaloric diet through a nasogastric tube. Despite the disease severity, normal growth, weight gain, and development were observed.

Because the child was refractory to the applied therapy, and massive proteinuria persisted with many complications, a unilateral nephrectomy was performed at the age of 7.5 months, and the remaining kidney was removed at the age of 9 months, and she started automated peritoneal dialysis.

**Figure 2:**
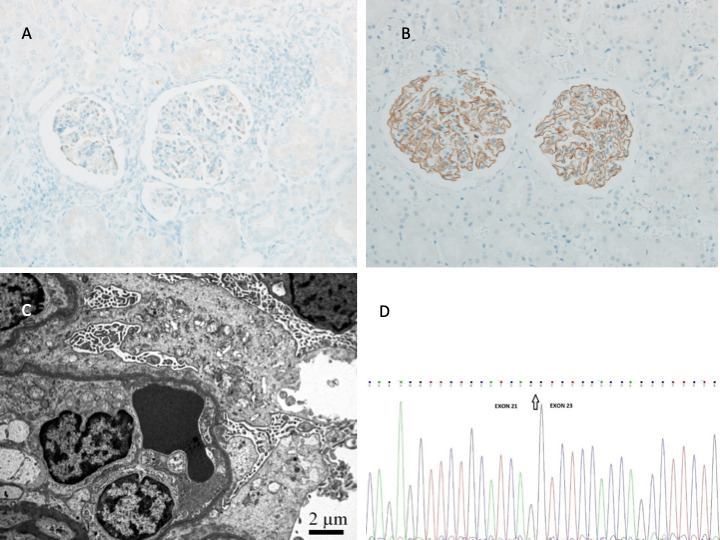
Negative immunohistochemical (IHC) stain for nephrin in a kidney of a child with Finnish-type congenital nephrotic syndrome with a *NPHS1* mutation, 200 × (**A**). Positive control for IHC stain for nephrin, 200 × (**B**). Diffuse podocyte foot process effacement with microvillous transformation on electron microscopy analysis (**C**). Sanger sequencing confirming the skipping of exon 22. Arrow indicates the splice site (boundary between exon 21 and exon 23) (**D**).

Histological examination of the resected kidneys revealed immature glomeruli showing diffuse mesangial sclerosis and mesangial hypercellularity. Some glomeruli were globally and segmentally sclerosed. Focal tubular microcystic dilatation and moderate interstitial fibrosis were observed. Immunohistochemical stain for nephrin was negative (Figure 2A,B). Electron microscopy study showed diffuse podocyte foot process effacement and an expanded mesangial matrix, with proliferation of mesangial cells (Figure 2C).

RNA was obtained from the kidney sample after nephrectomy from the patient, as well as from another unaffected patient as a control, and reverse transcribed into cDNA. In both patients, all except one cDNA fragment were appropriately long. In the affected patient, a homozygous shorter amplicon of *NPHS1* cDNA was observed, corresponding to exons 20-24. Both fragments were used for Sanger sequencing, which confirmed exon 22 skipping and revealed shorter translated cDNA (985 amino-acid long protein, instead of 1241) (Figure 2D). This is the first confirmation that this mutation affects cDNA and, consequently, protein translation. We additionally used the Human Gene Mutational Database to search for this mutation. It was found that substitution of C for G at c.2928-3 (which takes place in the case of deletion of C) is a disease-causing mutation that is predicted to induce a large splicing change.

At the age of 22 months, the patient received a deceased-donor kidney transplant (Table 1). One month after transplantation, donor-specific antibodies were identified in serum, which spontaneously decreased without specific treatment. One year and a half after transplantation, she is doing well, with normal renal function and no proteinuria detected.

**Table 1:** Medical history timeline

Year/age	Symptoms/signs	Diagnostic workup/diagnosis	Therapeutic intervention
2017/0 days (35 weeks of gestation)	weight 2520 g length 49 cm	/	/
2017/1 day	edema of the lower extremities	/	/
2017/3 days	edema of the extremities	proteinuria 4+ hypoalbuminemia (8 g/L) hyperlipidemia (cholesterol 8.3 mmol/L) hypogammaglobulinemia enlarged hyperechogenic kidneys on sonography	albumins parenterally (2 mg/kg/d) furosemide immunoglobulins every 14 d (0.4-0.5 g/kg) proteins in the diet (4 g/kg/d)
2017/1.5 months	anemia	severe neutropenia (378/μL) hemoglobin (7.9 g/dL) low erythropoietin level low thyroid hormone low antithrombin (3%; normal 83%-128%) low copper and ceruloplasmin level	copper up to 15 mg per day erythropoietin thyroid hormones acetylsalicylic acid feeding through a nasogastric tube
2017/2 months	/	genetically confirmed previously unreported homozygous variant c.2928-3del in the *NPHS1* gene	captopril (3 mg/kg/d) indomethacin (1 mg/kg/d)
2017/3 months	/	/	ibuprofen (3 mg/kg/twice per day) instead of indomethacin
2017/7.5 months	/	normal renal function proteinuria (4066 up to 11 656 g proteins per mol creatinine)	unilateral nephrectomy insertion of a catheter for automated peritoneal dialysis (APD)
2017/9 months	/	/	nephrectomy of the remaining kidney APD was started
2019/22 months	weight 12 kg	/	deceased-donor kidney transplant

## DISCUSSION

We reported on a patient with a typical clinical course of CNS, genetically confirmed as Finnish type with a novel homozygotic mutation in gene *NPHS1*. We observed not only massive proteinuria, severe hypoproteinemia, loss of immunoglobulins, and leakage of hormones but also severe neutropenia, which was attributed to low plasma copper and ceruloplasmin levels because the neutrophil level normalized after oral supplementation of copper, as already described (3).

In contrast to some reports on developmental delay and failure to thrive in patients with CNS (4), our patient experienced normal development, growth, and weight gain, which was very likely the result of early aggressive management.

Proteinuria did not significantly decrease after captopril and NSAID treatment, although some studies reported encouraging results in this regard (5,6).

Nephrotic syndrome did not recur in our patient. A recurrence of nephrotic syndrome soon after transplantation has been reported in 25% to 34% of patients with Finnish-type CNS, and anti-nephrin antibodies have been detected in the majority of patients with Fin-major homozygotes (7-9). In Fin-major homozygotes, the mutation stops gene reading very early, leading to a truncated protein of 90 amino acids as opposed to a truncated protein of 1109 amino acids in Fin-minor homozygotes and 1241 amino acids in a long normal nephrin molecule. Fin-major homozygotes have no immunological tolerance to nephrin, which leads to an increased probability of the development of anti-nephrin antibodies and a NS recurrence after transplantation. We showed for the first time that the mutation resulted in the lack of exon 22, as shown on a cDNA level, leading to a preliminary stop codon and truncated 985 amino-acid-long protein. Such a protein lacks a part of the intracellular component, similar to Fin-minor homozygotes. We do not therefore expect NS to recur in our patient (8-10).

Our patient is also heterozygote for the mutation in gene *FH*, for HLRCC. Such patients have an increased risk of renal carcinoma (10). However, our patient has already undergone bilateral nephrectomy.
